# Sexual Development in Non-Human Parasitic Apicomplexa: Just Biology or Targets for Control?

**DOI:** 10.3390/ani11102891

**Published:** 2021-10-04

**Authors:** Teresa Cruz-Bustos, Anna Sophia Feix, Bärbel Ruttkowski, Anja Joachim

**Affiliations:** Institute for Parasitology, Department of Pathobiology, University of Veterinary Medicine Vienna, Veterinaerplatz 1, A-1210 Vienna, Austria; Anna.Feix@vetmeduni.ac.at (A.S.F.); Baerbel.Ruttkowski@vetmeduni.ac.at (B.R.); Anja.Joachim@vetmeduni.ac.at (A.J.)

**Keywords:** apicomplexa, sexual reproduction, sexual stages, transmission blocking strategies, vaccination targets, *Cystoisospora suis*, *Toxoplasma gondii*, Plasmodium, *Eimeria*

## Abstract

**Simple Summary:**

Cellular reproduction is a key part of the apicomplexan life cycle, and both mitotic (asexual) and meiotic (sexual) cell divisions produce new individual cells. Sexual reproduction in most eukaryotic taxa indicates that it has had considerable success during evolution, and it must confer profound benefits, considering its significant costs. The phylum Apicomplexa consists of almost exclusively parasitic single-celled eukaryotic organisms that can affect a wide host range of animals from invertebrates to mammals. Their development is characterized by complex steps in which asexual and sexual replication alternate and the fertilization of a macrogamete by a microgamete results in the formation of a zygote that undergoes meiosis, thus forming a new generation of asexual stages. In apicomplexans, sex is assumed to be induced by the (stressful) condition of having to leave the host, and either gametes or zygotes (or stages arising from it) are transmitted to a new host. Therefore, sex and meiosis are linked to parasite transmission, and consequently dissemination, which are key to the parasitic lifestyle. We hypothesize that improved knowledge of the sexual biology of the Apicomplexa will be essential to design and implement effective transmission-blocking strategies for the control of the major parasites of this group.

**Abstract:**

The phylum Apicomplexa is a major group of protozoan parasites including gregarines, coccidia, haemogregarines, haemosporidia and piroplasms, with more than 6000 named species. Three of these subgroups, the coccidia, hemosporidia, and piroplasms, contain parasites that cause important diseases of humans and animals worldwide. All of them have complex life cycles involving a switch between asexual and sexual reproduction, which is key to their development. Fertilization (i.e., fusion of female and male cells) results in the formation of a zygote that undergoes meiosis, forming a new generation of asexual stages. In eukaryotes, sexual reproduction is the predominant mode of recombination and segregation of DNA. Sex is well documented in many protist groups, and together with meiosis, is frequently linked with transmission to new hosts. Apicomplexan sexual stages constitute a bottleneck in the life cycle of these parasites, as they are obligatory for the development of new transmissible stages. Consequently, the sexual stages represent attractive targets for vaccination. Detailed understanding of apicomplexan sexual biology will pave the way for the design and implementation of effective transmission-blocking strategies for parasite control. This article reviews the current knowledge on the sexual development of Apicomplexa and the progress in transmission-blocking vaccines for their control, their advantages and limitations and outstanding questions for the future.

## 1. Introduction

Cellular reproduction is a key part of the cellular (and organismic) life cycle. Both mitotic (asexual) and meiotic (sexual) cell divisions produce new individual cells. The general definition of sexual reproduction refers to the process where DNA combination of two cells creates new offspring through the fusion of two different cells (syngamy), nuclear fusion (karyogamy), and the final process of homologous chromosome crossover (meiosis). Sexual reproduction is widespread in all eukaryotic branches of the tree of life, and it is the predominant mode for recombination and segregation among all major groups of eukaryotes [1,2]. Sex evolved early in eukaryotic evolution, appearing approximately 850 million years ago, at least 200 million years after bacteria evolved [3]. The predominance of sexual reproduction in eukaryotes indicates that it has had a considerable success during evolution, and it must confer profound benefits, considering its significant costs relative to asexuality [4]. This success is due to meiosis, in which the ability to recombine the genomes of individuals passes a unique mixture of parental genes onto the next generation and makes selection highly efficient. Sex generates genetic variation, repairs DNA breaks and prevents accumulation of mutations [5,6].

The last eukaryotic common ancestor (LECA) had all of the features associated with modern eukaryotes: sexual reproduction through meiosis and genetic material inside a nucleus. It was thus capable of full “meiotic sex” [3,7]. Certain eukaryotes lost the ability to reproduce sexually, such as species of *Daphnia* and aphids [2,8], but ancient asexuality appears to be rare in eukaryotes [2,9]. The presence of genes encoding the meiotic machinery in early diverging protists implies that sexual reproduction arose early in eukaryotic evolution [7]. The term “protists” refers to those eukaryotes that are never multicellular, essentially any eukaryotic organism that is not an animal, plant, or fungus [10,11]. Certain lineages may be more closely related to animals, plants, or fungi than they are to other protists; however, the grouping is used for convenience, and the clade is divided in groups such as the SAR supergroup (Stramenopiles, Alveolata, and Rhizaria), Archaeplastida, Excavata (mostly unicellular flagellates), Amoebozoa, Hacrobia, Hemimastigophora, Apusozoa and Opisthokonta [12]. Sex is well documented in many of these groups; however, direct observations of sexual processes are missing for a majority of protist species, and in certain cases, entire protist lineages cannot be considered as “sexual” with any certainty yet. In many different taxa of parasitic protists, sex and meiosis are linked to transmission [8]. In certain well-studied clades from highly diverse lineages of amoebozoans, apicomplexans (Alveolata), and kinetoplastids (Excavata), sex is described to be induced under the (stressful) condition of having to leave their host, and either gametes or zygotes are transmitted to a new host and eventually disseminated which is key to the parasitic lifestyle [13]. In parasitic species that form cysts or oocysts, these resistant forms that are easily spread in the environment are often in the transmissible stages, and they may have occurred in early eukaryotes and may be linked to the evolution of sex [3,14].

The phylum Apicomplexa is a major group of protists, which includes species that cause important human and animal diseases worldwide. It is a monophyletic, extremely large and diverse group with more than 6000 named, and possibly, thousands of undescribed species. Five apicomplexan groups with medical and veterinary importance are gregarines, coccidia, haemogregarines, haemosporidia and piroplasms [12,15]. With few exceptions of non-parasitic (commensal or mutualist) species, Apicomplexa are obligatory parasites, and potentially every vertebrate and the majority of invertebrates host at least one species. It includes the causative agents of e.g., malaria, piroplasmosis, cryptosporidiosis, toxoplasmosis and coccidiosis. The genera *Babesia*, *Theileria*, *Cryptosporidium*, *Eimeria*, *Toxoplasma*, *Cystoisospora* and *Neospora* cause major diseases of veterinary importance linked to economic losses in the affected livestock. These parasitic diseases impair animal health, reproduction and growth and lead to economic losses with regard to decreased production, treatment costs, morbidity and mortality [16,17]. The most cost-effective strategies for the control, including prevention, elimination or eradication of pathogenic diseases are vaccines [3]. Compared to other pathogens such as viruses and bacteria, there is an eminent lack of antiparasitic vaccines; only two anti-nematode vaccines, one anti-tick vaccine and several antiprotozoal vaccines are commercialized for domestic animals [18]. Among all the antiprotozoal vaccines available, the anticoccidial vaccines for poultry are well developed. By contrast, no vaccine is available for protozoal diseases of humans [19,20], with the exception of two candidates for malaria, with promising results in early clinical trials [21,22,23].

Over the past decade, knowledge on the general biology of Apicomplexan parasites has increased considerably, and has helped to explore host-parasite interactions, vulnerabilities of important parasites within their developmental cycle, host immune reaction and development of immunity, all of which is of great importance for the development of control strategies. In addition, major advances in the current knowledge on parasite transmission biology has increased our understanding on the epidemiology and risk factors for infection. Apicomplexan parasites as a group infect a wide host range of animals from invertebrates to mammals (although the host range of a single parasite species can be restricted to a single host species). This development is frequently characterized by highly complex steps in the life cycle in which asexual and sexual replication alternate, and the fertilization of a macrogamete by a microgamete results in the formation of a zygote that undergoes meiosis, forming a new generation of asexual stages [24]. Depending on the taxonomic subgroup, development either is restricted to single or few host species, and the parasite concludes the whole endogenous life cycle in the same host species (e.g., in the coccidian genera *Eimeria* and *Cystoisospora*), or a host switch is required for the sexual development and completion of the life cycle (e.g., *Plasmodium*). The sexual stages constitute an important bottleneck in the life cycle of these parasites, as they are obligatory for the further development of transmissible stages. Consequently, the sexual differentiation process and the characterization of the gametocytes have become attractive targets for both basic research on cell biology and applied vaccine development. Sexual development of apicomplexan parasites leads to the production of gametocytes, which are characterized morphologically and by the expression of stage-specific genes; however, the initiation of gametogenesis is thus far only poorly understood [25,26]. Sexual development in different apicomplexan taxa varies greatly and still needs further research to provide sufficient details for translational and applied research [27].

Here, we review the current knowledge on the sexual development of Apicomplexa parasites and the progress in research on transmission-blocking vaccines for their control, their advantages and limitations, and possible approaches and outstanding questions for the future. 

## 2. Sexual Reproduction in Apicomplexa

**Figure 1:**
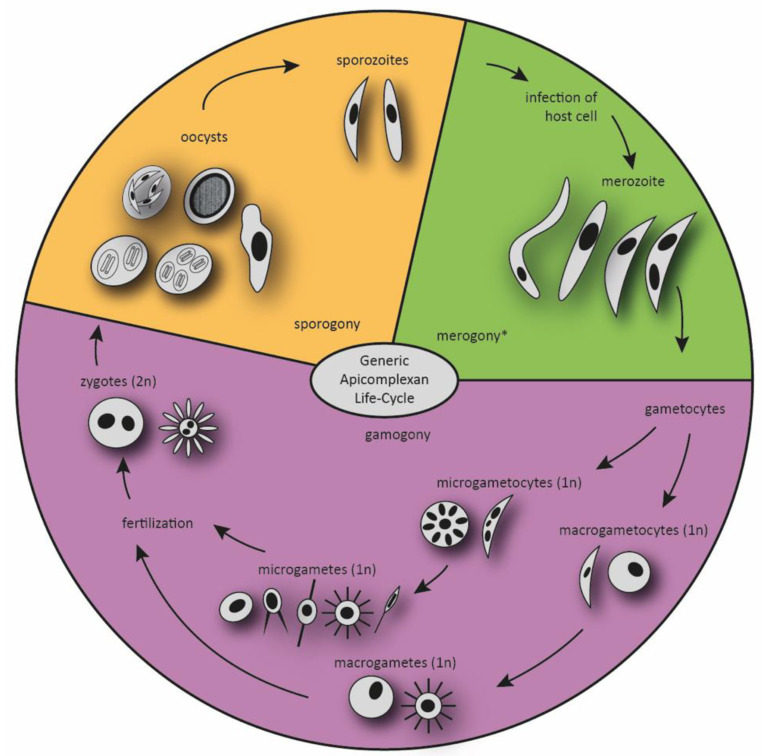
The generic life cycle of Apicomplexa, with a focus on gamogony. The apicomplexan parasites have complex life cycles that are characterized by three distinct processes: sporogony, merogony and gamogony. Transmissible stages are sporozoites, and in heteroxenous life cycles, merozoites or gametocytes. The various stages develop taxon-specifically in different forms and numbers. * In certain Apicomplexans, multiple merozoites will develop from meronts. After several rounds of merogony merozoites can develop into schizonts again, the parasite amount in the host animal can rise quickly. The different forms of all stages represent the variety of Apicomplexa developmental stages. Oocysts can be found in unsporulated and sporulated forms. The amount of nuclei varies regarding the parasite species and the parasite stage.

With few exceptions, Apicomplexa are obligatory parasites. Most members have a complex life cycle with significantly different forms (stages) characteristic for each of the main taxonomic groups of the phylum, and they fully depend on their hosts throughout most of their life cycle. The terminology used to describe these various life cycle stages varies between families, but the generic developmental cycle is divided into three distinct phases with alternating asexual and sexual multiplication—sporogony, merogony and gamogony—including four different basic cell types—sporozoite, merozoite, gametes (haploid types) and the zygote (diploid type) [24,28]. The release of sporozoites from the oocyst or sporocyst marks the initiation of the infection process, and host cells are invaded. The gliding sporozoite targets and invades the cells by using its eponymous apical complex, transforms into a trophozoite and immediately initiates a sequential series of asexual reproduction steps (referred to as merogony), resulting in the development of merozoites. These ultrastructurally resemble the sporozoite and infect further host cells, resuming asexual division to produce a new generation of merozoites. The merogonic cycle can be summarized as repeated events of invasion, replication and host cell egress of merozoites, and it precedes gamogony [29]. The final generation of merozoites is considered to be already sexually committed, despite morphological resemblance with the asexually reproducing preceding stages [30]. Although the trigger and the process of sexual differentiation of apicomplexan parasites are still obscure, they appear to be genetically programmed and not directly dependent on environmental changes [25]. In the following phase, the gamogony, some merozoites become female macrogametocytes (macrogamonts), while the majority transforms into male microgametocytes (microgamonts). The life cycle eventually proceeds to the fusion of a motile flagellated microgamete with a large and immobile macrogamete, with fertilization leading to the formation of a diploid zygote [28]. The apicomplexan zygote finally develops into the sporozoite. The process is termed sporogony and is characterized by cell divisions that can vary in numbers between taxa, with several rounds of meiosis and mitosis, resulting in the formation of infectious haploid sporozoites. Upon the transmission of sporozoites to the next host, the life cycle is completed (see Figure 1) [29,31].

The life cycles of different taxonomic groups vary in the number of hosts involved and the type of cellular invasion. In monoxenous species, the complete endogenous development occurs in a single host and frequently in a single cell type or tissue. By contrast, in heteroxenous species it involves different hosts and generally also different types of tissue. The life cycle of haemosporidia, piroplasms and coccidia is comprised of asexual multiplication, i.e., merogony and sporogony, and sexual development, gamogony [32,33], while most gregarines only practice gamogony and sporogony [34]. Although most Apicomplexa exhibit this overall general life cycle, the details can vary between species as outlined in more detail in Table 1.

The phylum is divided into two classes, the Conoidasida with the subclasses Coccidiasina, Gregarinasina and Cryptogregarinasina (and a number of orders contained therein) and the Aconoidasida which includes the order Haemosporidia and *Piroplasmida* [15,28,35,36].

### 2.1. Class Conoidasida

#### 2.1.1. Subclass *Gregarinasina*

Gregarines are the primitive lineage of the Apicomplexa. They have monoxenous life cycles with several exceptions (e.g., *Nematopsis* spp. utilize two hosts, crustaceans and mollusks) [37]. Most cycles comprise exclusively gamogony and sporogony, only few species include merogony [38]. Many gregarines do not exhibit intracellular stages and the sexual and asexual cycles occur extracellularly in the intestinal and coelomic cavities of their invertebrate hosts, setting the gregarines apart from most other apicomplexan taxa which infect vertebrates. The life cycle of most gregarines starts with the ingestion of the sporocyst by the host from which sporozoites are released and transform to trophozoites that attach to the host cell. Eugregarines infect the intestines, coeloms, and reproductive vesicles, neogregarines infect mainly the host tissues and archigregarines infect the intestines of the host [38]. There, the trophozoite increases in its size and consequently breaks the host cell. The developmental step that precludes gregarine sexual reproduction is called syzygy, the association of two mature trophozoites after they detach from the host cell. The two assembling cells are committed to develop into male and female gamonts, both unflagellated. Mature gametes are generally similar in shape and size and are produced in equal amounts [28]. Micro- and macrogametes mainly pair end-to-end before they fuse to form a zygote, but some have species-specific orientations such as head-to-head, tail-to-tail or head-to-tail [39]. The zygote forms a protective envelope, which later becomes the oocyst wall. Finally, it will undergo meiosis to produce new sporozoites, which will infect a new host [34,40]. Archigregarinida and Eugregarinida do not display merogony, while different types of merogony are reported in Neogregarines [39,41]. Although there is abundant literature about their life cycle, morphology and ultrastructure, gregarines are almost unstudied at the genomic/transcriptomic levels and have been the subject to only few biochemical analyses [40].

#### 2.1.2. Subclass *Cryptogregaria*

In contrast to the poorly studied gregarines, genomic studies applying a variety of molecular tools have greatly advanced our understanding of the biology of the genus *Cryptosporidium* [42], including sexual development. This genus was recently classified as the sole member of the new subclass *Cryptogregaria*, as it resembles the gregarines in many developmental aspects [43,44,45]. *Cryptosporidium* has a monoxenous life cycle consisting of several sexual and asexual stages. In *Cryptosporidium,* merozoites arising from type-II-meronts multiply sexually to produce gamonts. Macrogamonts develop further into large single-celled immobile macrogametes, whereas sixteen smaller single-celled microgametes without flagella develop from a microgamont, which bursts open to release the mature microgametes. Since 2002, when the first study by Hijjawi and colleagues demonstrated the presence of gamont-like extracellular stages in the life cycle of *Cryptosporidium*, several investigations tried to elucidate the origin of these stages [46]. To achieve fusion of micro- and macrogametes, both must appear at the same time and be compatible with each other. The microgametes fertilize the macrogametes, producing zygotes, which mature into oocysts [47]. During sexual differentiation, the expression of genes responsible for fundamental cellular processes changes widely. *Cryptospoiridium parvum* microgametes express the homologue of hapless2, a class II membrane fusion protein that is required for gamete fusion in Apicomplexa, similar to most eukaryotic organisms [48,49]. Macrogametes show an increased expression of conserved eukaryotic factors of meiotic recombination (DMC1, Spo11, HORMA and HOP2), meiosis-associated DNA repair, cell cycle regulation factors and genes involved in oocyst wall formation and wall persistence [50,51]. The oocyst wall forming protein COWP1 is expressed both in macrogametes and oocysts of *C. parvum* [52], and similar homologues also exist in other Apicomplexa [53,54,55].

#### 2.1.3. Subclass *Coccidiasina*

The order Eucoccidiorida includes a large number of pathogens of vertebrates with varying life cycles, host specificities and host roles [28], but all undergo sexual reproduction within specific hosts. However, the two suborders Adeleorina and Eimeriorina differ greatly in their life cycles; Adeleorina develop via syzygy while Eimeriorina produce independent gametes. Of the many genera and species of the order, the sexual development has been studied in only several, including the model species *T. gondii* [56,57,58], certain species of the genus *Eimeria* [25,59,60,61], and recently, *Cystoisospora suis* [53]. Sexual development of *T. gondii* occurs exclusively in the feline intestine [62], while *Eimeria* species can display distinct host and tissue tropisms, such as small and large intestines, stomach, the gallbladder or bile ducts, liver or gonads [63,64,65,66], but in most species, gamonts are formed in the intestinal lumen of the definitive host after merogony is completed, and the final generation of merozoites is considered to be sexually committed [67]. The early gamonts are already differentiated into micro- and macrogamonts, which are both immobile and morphologically similar in shape and size [67]. In the microgamont, the nucleus divides several times, and the newly formed nuclei locate to the periphery of the cell. Microgamete morphology varies among the Coccidia. The larger, unflagellated and immobile macrogamete contains a single nucleus larger than the small nuclei that are formed in the microgamont. The number of micro- and macrogametes that are formed from the gamonts varies greatly between coccidian taxa, but microgametes are usually greater in number than macrogametes [68,69]. The microgamete approaches the macrogamete and upon fusion of the two cells a zygote is formed. This is followed by several divisions of the zygote and simultaneous formation of the oocysts wall. The oocyst is the environmentally resistant stage that is either retained in the final host or discharged and disseminated in the environment [67,70].

*Eimeria* spp. complete their whole life cycles in the same host, and most species develop in the epithelial tissue of intestinal and adjacent (e.g., bile duct) tissues [71,72]. After a genetically fixed and taxon-specific number of merogony cycles [25,73], two distinct sexual stages occur [24]. Macrogametes are immobile and remain intracellular, whereas the biflagellated microgametes can move freely to find a compatible macrogamete [74,75]. The egress process of microgametes from host cell and fertilization still must be elucidated, only few studies show microgametes in contact with macrogametes [61,76], and since microgametes have not cell-invading machinery, this could suggest it occurs extracellularly in the gut lumen [25,54]. *Eimeria tenella* transcriptomic analysis showed an upregulation of over 800 transcripts involved in gametogenesis, identifying numerous sexual stage-specific genes. Many of these transcribed genes are intrinsically involved with gamont biology, related to axoneme and flagellum formation, locomotion, gamete membrane fusion, DNA condensation and oocyst wall formation [25,54,77].

In *Toxoplasma gondii*, macrogametes have an oval shape, a single nucleus, endoplasmic reticulum, mitochondria, lipid bodies, amylopectin inclusions, and two types of wall-forming bodies. Microgametes are elongated; they display a condensed nuclear chromatin, two flagella in the anterior region, and a mitochondrion located at the base of the flagella [78,79]. The fusion of micro-and macrogamete leads to the formation of an oocyst that is released into the intestinal lumen of the definitive host. Proteomic and transcriptomic studies uncovered hundreds of genes expressed uniquely in gametes, suggesting their potential role in gametocyte biology [78]. Recent studies can also show that upregulated genes in the sexual stages of *T*. *gondii* and *C. suis* (which is closely related to *T. gondii* and has sexual stages of similar morphology [80,81]) are those coding for proteins already highlighted as playing critical roles in the sexual biology of other Coccidia, including oocyst wall formation, microgamete motility and fertilization [56]. In *Toxoplasma*, *Cystoisospora*, *Eimeria* and *Cryptosporidium*, genes coding for oocyst wall proteins are already upregulated in macrogamonts, while proteins involved in axoneme-flagellar formation and motility and fusogen proteins are restricted to microgametes [82,83,84]. *Cystoisospora suis* also produces gamonts which develop further into clearly differentiated spherical macrogametes and microgametes. The flagella in *C. suis* are positioned on opposite sides, which might also affect microgamete movement on the search for a macrogamete whereas the flagella in *Eimeria* or *Toxoplasma* present a different morphology where the flagella are localized in the anterior region [25,78,85]. After in vitro merogony in epithelial host cells, *C. suis* also can continue gamogony in a host cell-free environment, indicating that gamete production and fusion occur extracellularly, as previously indicated for *C. parvum* [47,86]. 

The general development pattern of the suborder Adeleorina is highly complex, and at the species level, details in the life cycles often greatly differ [35,87]. All species in this suborder from gametes by syzygy. This involves the association of (often motile) gamonts prior to the formation of functional gametes and fertilization [28]. Despite this resemblance with the gregarines, Adeleorina are currently classified as Coccidia, and have similarly differentiated life cycles [88,89]. Members of the family Haemogregarinidae (common heteroxenous parasites of cold-blooded, less frequently warm-blooded vertebrates [90]) are characterized by their ability to invade different organs, cell types and hosts. They develop two morphologically different forms of meronts (micro- and macromeronts), which mainly infect erythrocytes and develop in these cells to sausage-shaped gamonts which subsequently give rise to differentiated micro- and macrogamonts, lying in syzygy in the same host cell. Macrogametes are larger and often immobile, whereas microgametes seem to be smaller, but occur in larger numbers. Haemogregarinidae show a large morphological diversity in microgametes, from unflagellated, monoflagellated, and biflagellated forms. Fusion of gametes (which occurs in invertebrates as definitive hosts) leads to formation of sporozoites enclosed in a sporocyst surrounded by an oocyst for continuation of the life cycle [90]. In contrast to Haemogregarinae, intracellular gametocytes of *Hepatozoon* (as a member of the Hepatozoidae) are found in the blood circulation either in erythrocytes or in leukocytes [91,92], and in all *Klossiella* species described to date, gametogony and sporogony occur in renal tissues [35].

### 2.2. Class Aconoidasida

#### 2.2.1. Order Haemosporida

In vertebrates, haemosporidian parasites form gamonts in blood cells after asexual development [28,89,93], either in tissue (families Haemoproteidae and Leucocytozoidae), in tissues flowed by erythrocytes (family Plasmodiidae) or in leucocytes (family Garniidae) [94,95]. When the infected blood cells of the vertebrate host are ingested by an arthropod, the immature gamonts develop into mature gametocytes. Microgametes often have up to eight flagella, whereas macrogametes are immobile. The fertilization is extracellular, the zygote (termed ookinete due to its motile nature), elongated and mobile, penetrates the epithelial gut until reaching the basal lamina, and the oocysts are formed [96]. After one or more sporogonies, infective sporozoites are formed, which are injected into the vertebrate host when the hematophagous arthropod feed [97,98]. The most representative genus of this order is *Plasmodium*. Malaria parasites display asexual reproduction twice in the vertebrate host, in liver hepatocytes (pre-erythrocytic merogony) and in red blood cells (blood-stage merogony), and once in the mosquito (sporogony) [99]. The essential sexual stage occurs at the transmission from vertebrate to insect. Certain asexual erythrocytic parasites develop into either male or female gametocytes within erythrocytes. These newly formed gametocytes undergo gametogenesis within the midgut of the mosquito where fertilization occurs [100]. The mature gamete types must fuse to form an ookinete, which penetrates the epithelial layer of the midgut of the mosquito to form an oocyst [98,101]. *Plasmodium falciparum* does not have chromosomes that determine the sex of the gametocytes, but all merozoites derived from a single committed meront become either micro- or macrogametocytes. Generally, more macrogametocytes than microgametocytes are produced. *Plasmodium* shows sexual commitment before the appearance of gametocytes [26]. Gametocyte sex allocation is therefore not only regulated by early stage-specific transcriptional factors, but also by protein phosphorylation [102]. Sexual commitment in *P. falciparum* is a well-described process. Recent work has shown that sexual differentiation is controlled by the expression of a master regulator, the transcription factor AP2-G [103]. Sexually committed cells are thereby allocated to the gene expression underlying sexual differentiation, by the activation of a transcriptional feedback loop that drives AP2-G expression up [27,104,105]. Other genes of the ApiAP2 family are the major transcript factors in *Plasmodium* development and especially HP1, HDA2 are also responsible for the epigenetic regulation during sexual commitment [27]. Activation of *AP2-G* expression triggers expression of early gametocyte genes including Pfs16, Pfg27/25, Pfg14.744, Pfg14.745 and Pfg14.748 [106]. HAP2/GCS1, P16, P48/45, P230, and FACT are transcribed in microgametes in *Plasmodium.* In the macrogametes, PKG and P47 are the main transcribing factors [98]. Furthermore, the homeodomain protein 1 (HDP1) was found to be an essential regulator of gene expression during the sexual differentiation of *Plasmodium*. HDP1 binds DNA during *Plasmodium* development and is tightly associated with chromatin in early gametocytes [107].

#### 2.2.2. Order Piroplasmida

Piroplasms are characterized by the eponymous pear-shaped intracellular stages in host blood cells. As mentioned above, merogony can occur in a variety of cells, and gamogony and sporogony occur in the gut and salivary glands of arthropods [28,108]. After merogony, rupture of host blood cell releases the merozoites which can undergo an undetermined number of rounds of merogony in the blood. Asexual division continues until merozoites can transform into gamonts [109]. Similar to *Plasmodium*, sexual multiplication of the parasite starts by gametocytes appearing in the host’s red blood cells. During the blood meal of the tick host, gametocytes develop into gametes that mature in the tick midgut lumen [110,111]. Macrogametes are large spherical immobile cells, whereas mobile microgametes form the families specific “spiky-rayed-stages”, which are sometimes referred to as pseudopodia-bearing gametes. *Theileria* spp. have anisogametes clearly distinguishable by light microcopy, unlike in *Babesia* spp. which do not differentiate into micro-and macrogametes, but equally formed isogametes, but still form two gametes populations, which differ in their shape and cytoplasm density [112,113,114]. During gamete fusion, a filamentous structure between gamete membranes is formed and subsequently a finger-like protrusion of one gamete penetrates the opposite one [109]. Gamete fertilization then gives rise to a zygote that penetrates the tick peritrophic matrix of the tick’s epithelial cells. Inside these, the zygote undergoes a meiotic division and results in the formation of kinetes, which are released into the tick’s hemolymph. The kinetes of *Theileria* spp. directly invade salivary glands (primary kinetes), while those of *Babesia* spp. parasites are subjected to two series of asexual multiplication in various tick tissues, and subsequent secondary kinetes invade the tick salivary glands [115]. Sporogony starts after kinete invasion of salivary gland cells, and the sporont is formed which has a polymorphous syncytium. The sporont later evolves into a multinucleated meshwork referred as a sporoblast, which is dormant during tick ecdysis. Maturation of the parasite sporoblast starts after tick attachment to the host and results in sporozoites being released into tick saliva [108]. Little is known about the expression of sexual stage-specific genes in piroplams. However, genes of the ApiAP2-family show changes in their expression level during the merogony of *T. annulata*. It hints to an involvement in the transmission of *T. annulata* to the tick vector, hence beginning gametogenesis [116]. An analysis of regulatory domains responsible for life cycle transitions in *Babesia bovis*, *Babesia microti* and *Theileria equi* show a high level of conservation between species and indicate that the transcriptional factor Api-AP2 is involved in the transition of the merozoites to gamonts [117]. Furthermore, *Babesia bovis* lacks 6-Cys sexual-stage genes, normally important for the development of sexual stages [118]. 

## 3. Breaking the Cycle—Targeting Sexual Stages of Apicomplexan Parasites for Intervention

In most apicomplexan species of medical and/or veterinary relevance, sexual differentiation produces dimorphic sexual stages, male and female gametes, those will fuse and for some, the fertilization culminates in the development of an oocyst [24]. Only a small proportion of asexual stages (merozoites) will differentiate into gametocytes, and therefore this step is to be considered a bottleneck of development—not only in *Plasmodium* [48,119] but in all Apicomplexa that produce gametocytes. The gametocytes are the transmissible stages to invertebrate definitive hosts (for blood-dwelling Apicomplexa) or leads to oocyst formation (for gut-dwelling Apicomplexa) [24,28]. In each developmental step, a reduction of the population size occurs, also from the formation of gametocytes to oocyst development, resulting in low numbers of infective parasites compared to the multiplying endogenous stages preceding them. For example, in *Plasmodium*, it has been estimated that out of the thousands of gametocytes that a female *Anopheles* mosquito typically ingests with a blood meal, only 50–100 develop into ookinetes and only several of them survive to form the infected oocysts. The lowest parasite numbers occur during the oocyst stage, but when each oocyst releases its sporozoites and merogony is initiated, the number of parasites again drastically increases. For this reason, apicomplexan sexual stages constitute good targets for vaccination, since their numbers are lowest in comparison to the other stages that develop throughout the parasite’s life cycle, and consequently, they are easier targets for the immune system [101]. As stated previously, the life cycles of the most important Apicomplexa are elucidated; however, the process of differentiation into the sexual stages or the fertilization process remains largely unknown, and such information would be essential to develop and implement control strategies that suppress the passage of parasites from one host to the next. Among the many approaches to control protozoan diseases, transmission-blocking strategies, developed to interrupt the life cycle using different mechanisms for halting transmission to the vertebrate hosts, appear to be the most logical type of prevention. To achieve a transmission blocking strategy, it is essential to consider the key elements of the parasite’s life cycle and to identify its weak points. Such strategies can be based on different mechanisms; one is the generation of transmission blocking vaccine with the induction of high antibody titers against the specific proteins of sexual stages. These antibodies will bind to the surfaces of the gametes to prevent/block the progression of parasite development. They do not directly protect immunized individuals but specifically block the transmission [120]. Another strategy is the identification of chemotherapeutic transmission blocking drugs that affect specifically the sexual stages [121,122]. A reduction in gametocytes can result in reduced transmission, and both transmission-blocking vaccines and transmission-blocking drugs may have the potential for eradication of a disease. In recent years, the accessibility to the transcriptomes and proteomes of sexual stages provides a useful tool and support the definition of sex-specific gene transcripts and protein candidates for vaccination, some of which are exclusively expressed in either immature or mature gametocytes [25,48].

Coccidia are characterized by the environmental oocyst as the final result of the fusion of gametocytes; thus, the reduction of this final stage would lead to a decrease in environmental contamination, and with this, the infection of new hosts. Interruption of the parasites’ life cycle before the formation of oocysts has been shown to abrogate oocyst formation. Over the past 40 years, the precise knowledge of the sexual reproduction of *Eimeria* genus resulted in the commercialization of a transmission-blocking vaccine against different *Eimeria* species in order to prevent coccidiosis in chickens. Wallach and coworkers, in the 1980s, were the first group to report immunization of chicken with *Eimeria maxima* gamont-specific proteins recognized by serum IgY collected from chickens that had recovered from *E. maxima* infection. In following studies, two glycosylated tyrosine-rich proteins of the wall-forming bodies of *E. maxima*, 56 and 82 KDa in size, were identified as antigens conferring protection because of a strong serum antibody response, not only against *E. maxima* but also against other *Eimeria* species of chicken due to their high degree of conservation across the genus [123,124,125,126]. In 2002, the commercial vaccine CoxAbic^®^ using the native gametocyte antigens of *E. maxima*, Gam56 and Gam82, was commercialized. In clinical trials, CoxAbic^®^ reduced oocyst shedding of the three major species of *Eimeria* (*E. maxima, E. tenella,* and *E. acervulina*) in broiler chicken by 50–80%. The assumption was that the vaccination with these gam proteins stimulated an immune response that blocked the construction of the oocyst wall [127]. The production of this vaccine based on affinity purification of native gametocyte proteins from parasites, however, was expensive and time-consuming. Since then, several proteins (molecular weights of 14, 22, 30, 56, 82 and 230 kDa) associated with *E. maxima, E. tenella,* and *E. necatrix* gametocytes have been proposed as potential vaccine targets to induce immunity [128,129,130,131,132,133]. The most recent study using Gam proteins involve the recombinant rEtGam22. Vaccination of chicken with EtGam22 significantly elicited both Th1 and Th2 cytokine mediated immune responses and induced protection against *E. tenella* and *E. maxima*-infected chickens [129]. Taken together, these studies indicate that Gam proteins are potent immunogens for the use as vaccines against chicken coccidiosis, as they induce a diverse and robust immunity [134].

Regarding male gametocytes and their role in protection, monoclonal antibodies against microgametocytes of *E. tenella* reduced the oocyst formation in vitro more than 50% but the mechanism of inhibition was not investigated in detail [60], and this approach was not followed further. Oral application of sera containing *E. tenella* gamont-specific monoclonal antibodies significantly reduced oocyst output and cecal lesions in passively immunized chicken but the direct role of fertilization inhibition in immune-mediated protection was not explored further [135]. A new approach in the era of genetic manipulation technologies is the production of knockout (KO) parasite strains to be used as genetically attenuated live vaccines [136]. Recently, a HAP2-deficient *T. gondii* strain was created using the CRISPR/Cas9 approach. HAP2/GCS1 is a male fertility factor; it is a conserved protein expressed in male gametocytes, which was originally identified in *Arabidopsis thaliana* and later in *Plasmodium* spp. [49,137,138]. The HAP2-deficient *T. gondii* generated parasites that could infect cats and start asexual multiplication but failed to complete fertilization and undergo meiosis, which completely inhibited oocyst formation and excretion. Inoculation with HAP2-deficient *T. gondii* tissue cysts to cats completely prevented oocyst excretion, simultaneously inducing immunity to oral challenge with tissue cysts from wild-type *T. gondii*. This demonstrated impressively that interfering with fertilization can completely block transmission of this parasite and that the development of a transmission-blocking vaccine is feasible for coccidia [56].

Independent of oocyst formation, gamete formation is also crucial for the development of downstream infectious stages (sporozoites) in Haemosporida and Piroplasmida. In *Plasmodium*, numerous sexual-stage specific proteins with antigenic properties and novel enzymes that putatively regulate functions during sexual-stage development have been suggested as candidates for vaccination or drug targets. Different surface proteins of macrogametes, such as Pfs48/45 and Pfs230 (both playing a role in male gamete fertility) have been shown to be immunogenic [139]. Zygote surface proteins expressed only post-fertilization in the mosquito host, such as Pfs25 and Pfs28, have also been considered as antigen candidates for transmission-blocking vaccines. In animal studies, Pfs25 vaccine candidates induced equal or greater serum transmission-blocking activity compared to other antigens or antigen combinations, and hence Pfs25 has been the focus of clinical trials published to date [139,140]. Ongoing trials are now examining the activity of Pfs230 and other transmission-blocking vaccine candidates, and it is expected that at least some of them will enter the clinical phase in the coming years [141,142]. Other studies proposed HAP2 protein as a candidate for a transmission-blocking vaccine in *Plasmodium*. When the hap2 gene is absent or mutated, the zygote formation is completely blocked, indicating its relevance in this event [143]. Antibodies targeting HAP2 inhibited *P. berghei* transmission in vivo by 58.9%, and anti-PvHAP2 antibodies reduced infection prevalence by 50% in *P. vivax* [144,145]. In addition, the glycolytic pathway has been proposed as a target for intervention as it is the source of energy for flagellar beat and hence mobility of the microgamete [146]. Motility through flagellar action is a unique feature of this stage [74], and the flagellum was proposed as a target in earlier works on immune intervention in *Eimeria* [135] and in *Plasmodium* [147,148]. In addition, a range of kinases, surface proteins, signaling and regulatory proteins of *Plasmodium* involved in sexual stage development, differentiation and zygote formation have been proposed as candidates [139]. In the piroplasm *Babesia*, specific antibodies against HAP2 significantly decreased zygote formation in vitro [149,150].

## 4. Concluding Remarks

Diseases caused by apicomplexan parasites, both in domestic animals and in humans, represent a significant health and economic problem in affected populations. Despite the availability of treatment against many of these diseases, there is an urgent need for vaccination strategies due to poor drug efficacy (mostly during the early stages of the infection), serious side effects of treatment, and the increasing resistance of parasites against parasiticides [20,151]. Considering these issues, a preventive vaccine appears essential for sustainable disease control. Vaccinating against apicomplexan parasites, however, is a complex task. The difficulties in the development and implementation of such vaccines include the intriguing complexity of the parasites’ life cycles, involving different hosts, stages and affected tissues, and the existence of poorly accessible arthropod vectors in many species, but also the lack of in vitro culture technologies and suitable in vivo animal models for large-scale screening. In certain taxa, the life cycle is not known in sufficient detail to study effects of intervention on parasite development. An additional factor is that the complex interactions between the parasites and the host’s immune system have co-evolved to create (at least in most cases) a balance between host and parasite. As a result the apicomplexan protozoa are frequently capable of evading host immune responses by targeting privileged intracellular sites, immune evasion mechanisms or antigenic variation during its development [19].

Reproduction is critical for perpetuating a species; however, the modes by which cell division can occur are diverse. Fusion of gametes of opposite sex (or mating cell type) to form a zygote is the defining moment of the cellular sexual reproduction. With few exceptions, sexual reproduction appears to be a hallmark of apicomplexan development. During sexual reproduction, genetic recombination occurs, having a significant impact on parasite genomics. Recombination almost always produces novel genotypes, which ultimately results in an increase in genetic variability that may enhance the fitness of the parasites in an ever-changing environment (e.g., facilitating evasion of the host’s immune system and accelerating the spread of mutations conferring resistance to drugs), and reduces the accumulation of deleterious mutations. Previously published studies already proposed that targeting the sexual stages should primarily inhibit the formation of fertile oocysts thus acting as a transmission-blocking vaccine [25]. For example, using the Gam proteins as vaccination to target the oocyst wall formation in *Eimeria* reduced oocyst shedding in the host, and the vaccines that incorporate surface antigens of the macrogametes of *Plasmodium* in order to induce antibodies that kill parasites in the mosquito bloodmeal and interrupt parasite transmission through the vector significantly reduced oocyst formation. Monoclonal antibodies against microgametes of *E. tenella* could reduce oocyst formation [60], HAP2 KO parasites fail to complete fertilization and zygote formation in *Toxoplasma* [56] and antibodies against HAP2 significantly decreased zygote formation in vitro [144,149,150]. Current research on transmission-blocking vaccines seeks to increase the degree and the durability of functional activity through the combination of different antigens, engineered immunogens, and the optimization of vaccine delivery system and adjuvants. It is required to achieve a sufficient adaptive response to maintain high levels of antibodies over time, as well as broad coverage of antigen variations, to achieve immunity. In addition, transmission-blocking vaccines must have an exceptional safety profile, as they do not confer a direct benefit to the individual. Previous findings indicate that inhibiting either (or both) the fertilization of macrogametes by microgametes (which is necessary for the production of next-generation sporozoites) and the oocyst wall formation can effectively interfere with the parasite’s developmental cycle. Targeting such stages may well be an effective approach to apicomplexan parasite control in the future.

## Figures and Tables

**Table 1:** Overview of characteristic life cycle events of Conoidasida and Aconoidasida. ✔, present; ✕, absent; **?**, uncertain. Apicomplexan life cycles can vary greatly between taxa and species; however, shared characteristics are described here. ^a^, the absence of merogony is dependent on the species; ^b^, the two gamete populations are indistinguishable by light microscopy; ^c^, the mobility of the gametes is dependent on the species; ^d^, in Eugregarinorida this process is called “gamete-dance”, as both gametes move in circles around each other. The table is composed based on the references provided in the text.

Class.	Conoidasida						Aconoidasida	
Subclass	Gregarinasina			Cryptogregaria	Coccidiasina			
Order	Archigregarinida	Eugregarinida	Neogregarinida	Cryptogregarida	Eucoccidiorida		Haemosporida	Piroplasmida
Suborder					Adeleorina	Eimeriorina		
Heteroxenous	✕	✕	✕	✕	✔	✔	✔	✔
Monoxenous	✔	✔	✔	✔	✔	✔	✕	✕
Merogony	✕	✕	✕✔ ^a^	✔	✔	✔	✔	✔
Syzygy	✔	✔	✔	✔	✔	✕	✕	✕
Gamogony In	Invertebrates	Invertebrates	Invertebrates	Vertebrates	Vertebrates	Vertebrates	Invertebrates	Invertebrates
Spiky-Rayed-Stages	✕	✕	✕	✕	✕	✕	✕	✔ ^b^
Free Gametocytes	✔	✔	✔	✔	✔	✔	✔	✔
Differentiation of Gametes	✔	✔	✔	✔	✔	✔	✔	✕
Microgamete motile	✔	✔	✔	✔	✔	✔	✔	✕✔ ^c^
Macrogamete motile	✕	✕	✕	✕	✕	✕	✔	✔
Sexual Cycle Intracellular	?	?	?	✕	✔	?	✔	✔
Gametes Fusion	✔	✔ ^d^	✔	✔	✔	✔	✔	✔
Zygote Formation	✔	✔	✔	✔	✔	✔	✔	✔
Oocyst Formation	✕	✕	✕	✔	✔	✔	✔	✔
Final Product	Oocyst	Oocyst	Oocyst	Oocyst	Oocyst	Oocyst	Ookinete/Oocyst	Ookinete
